# Beneficial bacteria activate type-I interferon production via the intracellular cytosolic sensors STING and MAVS

**DOI:** 10.1080/19490976.2019.1707015

**Published:** 2020-01-15

**Authors:** Jorge Gutierrez-Merino, Beatriz Isla, Theo Combes, Fernando Martinez-Estrada, Carlos Maluquer De Motes

**Affiliations:** School of Biosciences and Medicine, University of Surrey, GU2 7XH Guildford, UK

**Keywords:** Lactic acid bacteria, beneficial microbes, interferon, STING, MAVS

## Abstract

Type-I interferon (IFN-I) cytokines are produced by immune cells in response to microbial infections, cancer and autoimmune diseases, and subsequently, trigger cytoprotective and antiviral responses through the activation of IFN-I stimulated genes (ISGs). The ability of intestinal microbiota to modulate innate immune responses is well known, but the mechanisms underlying such responses remain elusive. Here we report that the intracellular sensors stimulator of IFN genes (STING) and mitochondrial antiviral signaling (MAVS) are essential for the production of IFN-I in response to lactic acid bacteria (LAB), common gut commensal bacteria with beneficial properties. Using human macrophage cells we show that LAB strains that potently activate the inflammatory transcription factor NF-κB are poor inducers of IFN-I and conversely, those triggering significant amounts of IFN-I fail to activate NF-κB. This IFN-I response is also observed in human primary macrophages, which modulate CD64 and CD40 upon challenge with IFN-I-inducing LAB. Mechanistically, IFN-I inducers interact more intimately with phagocytes as compared to NF-κB-inducers, and fail to activate IFN-I in the presence of phagocytosis inhibitors. These bacteria are then sensed intracellularly by the cytoplasmic sensors STING and, to a lesser extent, MAVS. Accordingly, macrophages deficient for STING showed dramatically reduced phosphorylation of TANK-binding kinase (TBK)-1 and IFN-I activation, which resulted in lower expression of ISGs. Our findings demonstrate a major role for intracellular sensing and STING in the production of IFN-I by beneficial bacteria and the existence of bacteria-specific immune signatures, which can be exploited to promote cytoprotective responses and prevent overreactive NF-κB-dependent inflammation in the gut.

## Introduction

Type-I IFN (IFN-I) such as IFN-α and IFN-β are polypeptides produced by innate immune cells in response to infectious agents, particularly viral pathogens^1^. IFNs are important to induce antimicrobial responses in an autocrine and paracrine manner via the synthesis of interferon-stimulated genes (ISGs), and to modulate innate immune responses that result in a balanced natural killer cell response and effective antigen presentation to activate immunological memory through specific T and B cells responses. IFN-I can also display protective roles against bacterial infections, cancer and autoimmune diseases.^2^ Innate immune cells produce IFN-I upon recognition of microbe-associated molecular patterns (MAMPs) via pattern recognition receptors (PRRs). The first described PRRs were Toll-like receptors (TLRs) and Nucleotide-binding oligomerization domain (NOD)-like intracellular receptors (NLRs). These PRRs activate an intracellular signaling cascade that converges on the pro-inflammatory transcription factor nuclear factor kappa-B (NF-κB), which is crucial in regulating anti-microbial responses in the mucosa.^3^ The production of IFN-I requires the additional and concomitant activation of IFN regulatory factors (IRFs), which can be achieved via the TLR adaptors myeloid differentiation primary response 88 (MyD88) and TIR domain-containing adapter-inducing IFN (TRIF).^4^ More recently, the cytosolic molecules stimulator of interferon genes (STING) and mitochondrial antiviral signaling (MAVS) have been identified as potent IFN-I inducers in response to nucleic acids.^5^ STING is an endoplasmic reticulum-localized transmembrane protein that becomes activated in the presence of cytosolic DNA and the production of 2‘,3‘-cyclic GMP-AMP (cGAMP) by the cGAMP synthase (cGAS).^6–9^ In addition, STING can also recognize other cyclic dinucleotides (CDN) of prokaryotic origin.^6,10^ MAVS is a mitochondria-localized transmembrane protein that is activated by retinoic acid inducible gene (RIG)-I and melanoma differentiation-associated protein (MDA)-5, two cytosolic sensors of 5‘ tri- and di-phosphate RNA and stretches of dsRNA.^11–13^ Both STING and MAVS coordinate the activation of TANK-binding kinase (TBK)-1 and IRF3, which translocates into the nucleus to initiate IFN production.^14,15^ The role of STING and MAVS in inducing protective immune responses in the presence of cytosolic DNA and RNA such as those from viral infections or intracellular pathogenic bacteria is well known,^16,17^ and in addition, STING has recently been shown to mediate sensing and autophagy of live gram-positive bacteria.^18^ However, the impact of STING and intracellular sensing on immune responses mediated by gut microbes remains relatively unexplored.

Humans live in symbiosis with beneficial microbes that inhabit different parts of the body, including the gastrointestinal and respiratory tracts.^19,20^ Beneficial microbes are composed of commensal bacteria that are critical to maintain homeostasis due to their involvement in appropriate host immune responses. One of the most significant bacterial groups associated with host beneficial properties are lactic acid bacteria (LAB),^21^ typical commensals that play an important role in activating epithelial cells, antigen-presenting cells and phagocytes.^22^ LAB interact with innate immune cells via numerous MAMPs including pili (fimbriae), peptidoglycans, lipo-teichoic acids, exopolysaccharides and many other components of the cell wall and membrane,^22^ that are recognized extracellularly by TLR and NLR and trigger NF-κB responses.^21,22^ A few studies have also reported that LAB are capable of inducing protective pro-inflammatory responses that depend not only on NF-κB activation, but also on IRFs.^23,24^ The specific mechanisms that LAB utilize to activate IRFs are unknown, but it has been reported that the production of type-I interferons (IFNs) requires the presence of TLRs in endosomal compartments, including TLR2 and TLR3.^23,24^

Here we demonstrate that different LAB species induce specific immune responses in human macrophages and primary cells, and that those that activate IFN-I responses fail to trigger NF-κB activation. Contrary to current dogma in which beneficial bacteria are mostly sensed by TLRs, we show that IFN-I-inducing LAB are sensed intracellularly via cytosolic nucleic acid sensors and that IFN-I production and ISG induction is dependent on STING and, to a lesser extent, MAVS. Our findings provide a novel perspective into the interactions between gut microbes and human cells that have implications in the use of and manipulation of beneficial commensal bacteria to modulate human immune responses.

## Results

### LAB activate NF-κB or IFN-I responses in THP-1 macrophages

**Figure 1.**
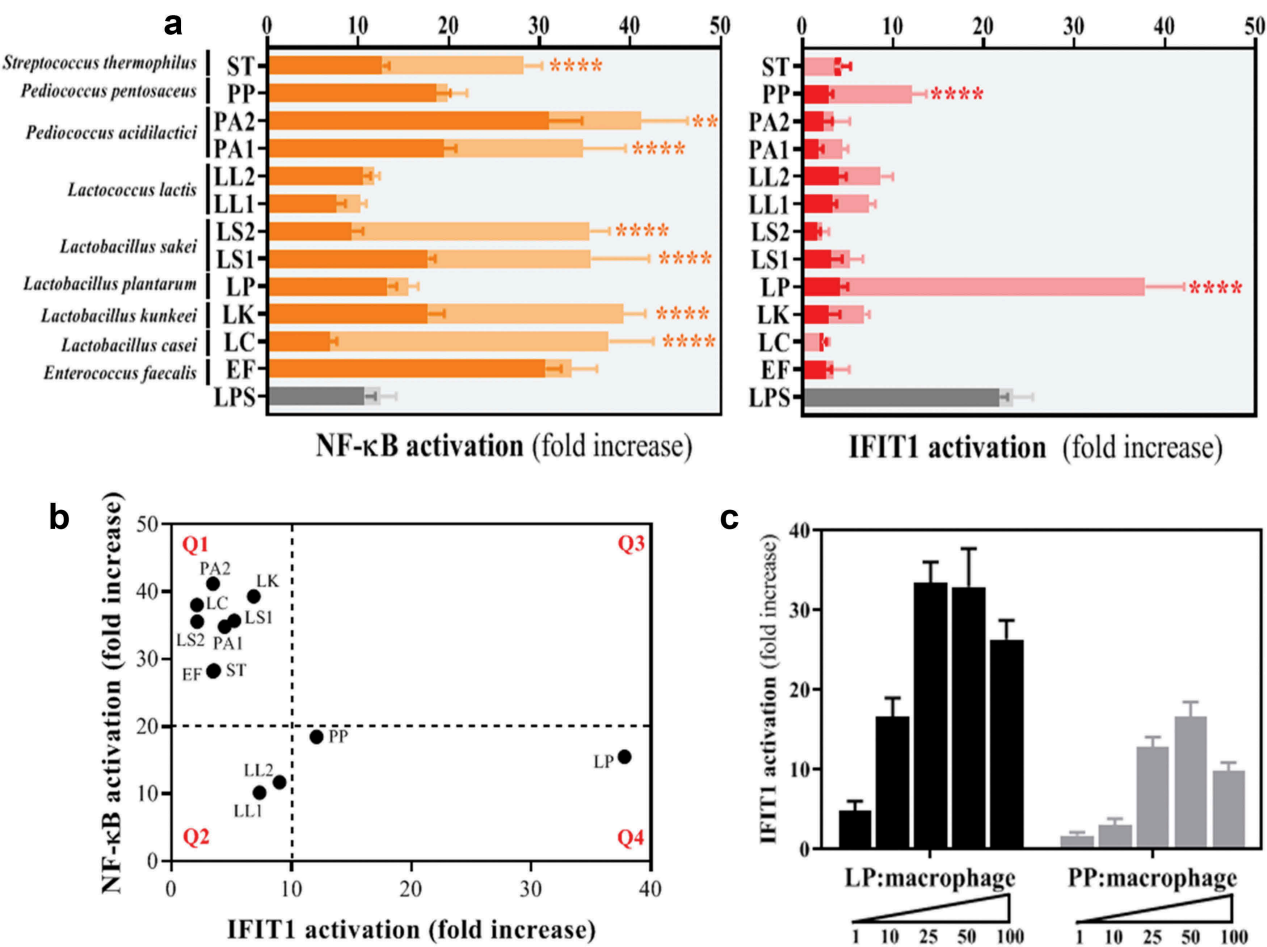
Analysis of 12 LAB strains reveals that viable cells of *Lactobacillus plantarum* (LP) and *Pediococcus pentosaceus* (PP) significantly induce IFIT1 activation in THP-1 macrophages in a dose-dependent manner. (a) Response of PMA-differentiated THP-1 macrophages to LAB isolates as a measurement of NF-κB (orange) and IFIT1 (red) activation. GLuc activation is presented as a fold increase over a non-stimulated condition. LAB were used at a ratio of 1 macrophage per 100 inactivated bacterial cells (dark orange and red) or 25 viable bacteria (light orange and red). LPS (gray bars) was used as a positive control in all screens. For each of the isolates, a comparative statistical analysis was carried out between inactivated and viable cells using the Student’s *t*-test (**p < .01; ****p < .001). (b) Correlation between NF-κB and IFIT1 activation in PMA-differentiated THP-1 macrophages exposed to LAB at a ratio of 1 macrophage per 25 viable bacteria. Activation is presented as a fold increase over a non-stimulated condition. Fold increases of 20 and 10 were selected as arbitrary thresholds for NF-κB and IFIT1 activation, respectively, to divide the graph into quadrants (Q1, Q2, Q3 and Q4). (c) IFIT1 activation in PMA-differentiated THP-1 macrophages challenged with viable cells of LP (black bars) and PP (gray bars) at increasing macrophage:bacteria ratios of 1:1, 1:10, 1:25, 1:50 and 1:100. The activation is presented as a fold increase over a non-stimulated condition.

The ability of 12 LAB strains (Table 1) to trigger the activation of the inflammatory transcription factors NF-κB and IRF-3 was evaluated in human differentiated THP-1 cells. We employed 2 lines of THP-1 monocytes expressing Gaussia luciferase (GLuc) under the control of either the NF-κB promoter or the promoter of the IRF3-dependent gene IFN-induced protein with tetratricopeptide repeats (IFIT)-1. The cells were differentiated for 48 h and subsequently exposed to live or dead LAB before GLuc activity was measured in the media and presented as a fold increase over non-stimulated macrophages (Figure 1(a)). The majority of LAB species tested – *Streptococcus thermophilus, Pediococcus acidilactici, Lactobacillus sakei, Lactobacillus kunkeei, Lactobacillus casei*, and *Enterococcus faecalis* - induced significant NF-κB activation and, with the exception of *E. faecalis*, this activation was enhanced in response to live bacteria compared to inactivated bacterial cells. By contrast, we found that these isolates were poor inducers of IFIT1 activation either dead or alive. Interestingly, two bacterial species – *Pediococcus pentosaceus* and *Lactobacillus plantarum* – were able to induce a significant IFIT1 activation. This IFIT1 response was only observed with viable bacteria and was especially high with *L. plantarum*. Interestingly, neither *P. pentosaceus* nor *L. plantarum* enhanced NF-κB activation when used as viable bacteria. To visualize this contrasting behavior, we then plotted the recorded NF-κB vs IFIT1 activation for each LAB species and drew arbitrary cutoffs for NF-κB activation (~20 fold) and IFIT1 (~10 fold) based on the most common response observed in all bacterial species tested (Figure 1(b)). As expected most LAB grouped in the top left quadrant (Q1) as powerful NF-κB agonists, but poor IFIT1 inducers. Strikingly, *L. plantarum* and *P. pentosaceus* appeared in the bottom right quadrant (Q4) as remarkably strong IFIT1 inducers, especially *L. plantarum*. The 2 *L. lactis* species occupied the bottom left quadrant (Q2) as intermediate NF-κB and IFIT1 inducers. We then performed dose-dependent exposures with *L. plantarum* and *P. pentosaceus* and showed that a challenge with 1 *L. plantarum* and 10 *P. pentosaceus* bacterial cells per macrophage was sufficient to trigger IFIT1 activation (Figure 1(c)). The highest IFIT1 response was observed with a ratio of 25 bacteria per macrophage. Our screening therefore indicated that some LAB could activate responses converging on IFN-I production.

**Table 1.** Lactic Acid Bacteria (LAB) isolates used in this study

Species	Strain	Internal code	Source	Reference	Donor/Culture collection
*Enterococcus faecalis*	NCTC 8213	EF	Human babies’ faeces	^25^	Surrey Culture Collection
*Lactobacillus casei*	I-1518	LC	Probiotic Drink	^26^	CNCM
*Lactobacillus kunkeei*	O29	LK	Bee pollen	This study	
*Lactobacillus plantarum*	WCFS-1	LP	Human saliva	^27^	Prof M Kleerebezem, Wageningen University, the Netherlands
*Lactobacillus sakei*	Lb790	LS1	Meat	^28^	Dr L Axelsson, Nofima AS, Norway
*Lactobacillus sakei*	L45	LS2	Norwegian fermented dried sausage	^29^	Prof I Ness, Norwegian University of Life Sciences
*Lactococcus lactis*	BBS4	LL1	Spanish fermented dried sausage	^30^	Prof PE Hernandez Cruza, Universidad Complutense de Madrid
*Lactococcus lactis*	IO-1	LL2	Kitchen sink water	^31^	Prof H Yoshikawa, Tokyo University of Agriculture
*Pediococcus acidilactici*	PAC1.0	PA1	Fermented food	^32^	Prof J Kok, Groningen University, the Netherlands
*Pediococcus acidilactici*	E1	PA2	Raw honey	This study	
*Pediococcus pentosaceus*	NCTC 990	PP	Dried beer yeast	^33^	Surrey Culture Collection
*Streptococcus thermophilus*	I-1613	ST	Yogurt	^34^	CNCM

### L. plantarum and P. pentosaceus activate IFN-I production

**Figure 2.**
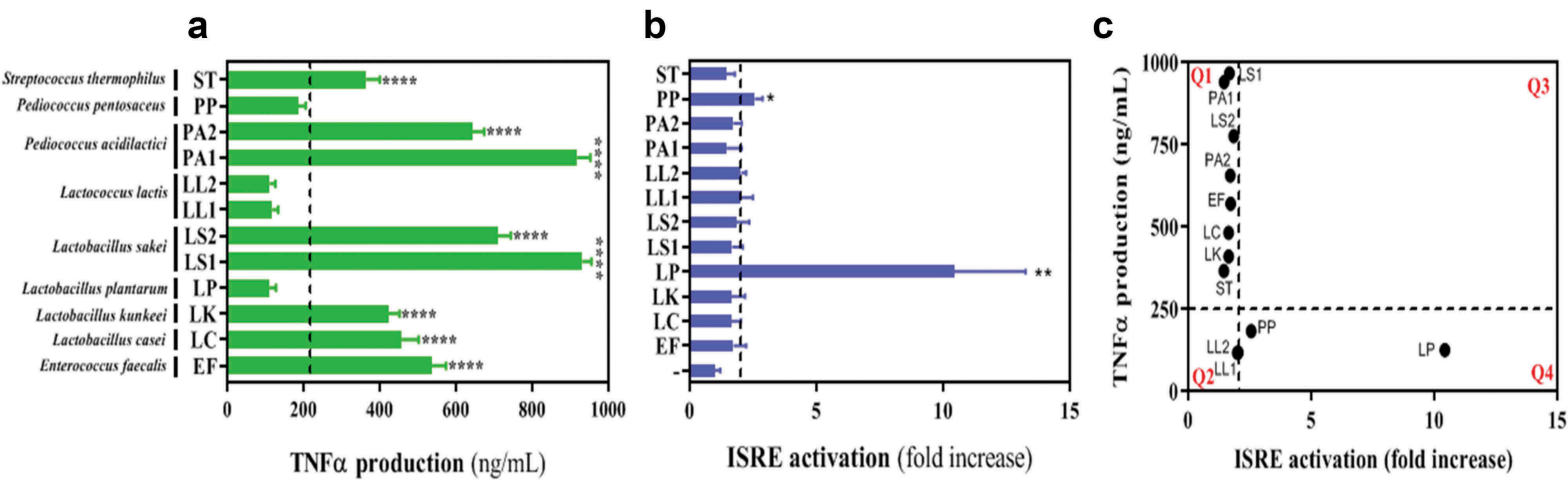
*Lactobacillus plantarum* (LP) and *Pediococcus pentosaceus* (PP) barely induce TNF-α production in THP-1 macrophages but significantly induce ISRE activation. (a-b) Supernatants obtained from THP-1 macrophages exposed to LAB isolates at a ratio of 1:25 were analysed for the presence of (a) TNF-α production using a commercial TNF-α ELISA and (b) ISRE activation using a pISRE-FLuc/RLuc bioassay. In both cases, isolates on the right-hand side of the dotted line induced statistically significant responses compared with the rest as determined by one way ANOVA followed by Fisher’s Least Significant Difference (LSD) Test (*p < .05; **p < .01; ****p < .001). (c) Correlation between TNF-α production and ISRE activation in THP-1 macrophages exposed to each of the LAB selected in this study at a ratio of 1:25. Doted lines indicate the thresholds used for TNF-α concentration (250 ng/mL) and ISRE activation (2-fold increase) to divide the graph into quadrants (Q1, Q2, Q3 and Q4).

To address expression of IFN-I and verify the activation observed using luciferase reporters, we used an IFN-stimulated responsive element (ISRE)-based bioassay and a commercial ELISA against the NF-κB-dependent inflammatory cytokine TNF-α to quantify the presence of TNF-α and IFN-I in the supernatants of macrophages exposed to each of the LAB isolates (Figure 2(a,b), respectively). Apart from *L. plantarum, P. pentosaceus* and isolates of *L. lactis*, the exposure of macrophages to all LAB isolates resulted in high amounts of TNF-α in the media, but very low levels of IFN-I. Conversely, *L. plantarum* and *P. pentosaceus* were capable of inducing a significant ISRE activation as indicative of effective production of IFN-I, especially in the case of *L. plantarum* (Figure 2(b)). Similarly to the luciferase-based results, when the production of TNF-α and IFN-I were plotted we identified a strong IFN-I signature for *L. plantarum* that correlated with a very low impact on TNF-α (bottom right quadrant Q4 of Figure 2(c)). The arbitrary cutoffs for production of TNF-α (~250 ng/mL) and IFN-I (~2 ISRE fold increase) were selected based on the most common response observed in all LAB tested. *P. pentosaceus* showed a more moderate IFN-I signature and a slightly higher TNF-α production than *L. plantarum* (next to the bottom left quadrant Q2 of Figure 2(c)), but this was still much lower than that observed with most of the LAB isolates (top left quadrant Q1 of Figure 2(c)). Taken together, our screening demonstrated that each LAB has the ability to stimulate different innate immune responses in human macrophages and that some are potent inducers of IFN-I.

### L. plantarum and P. pentosaceus activate responses associated with IFN-I production in human PBMCs

**Figure 3.**
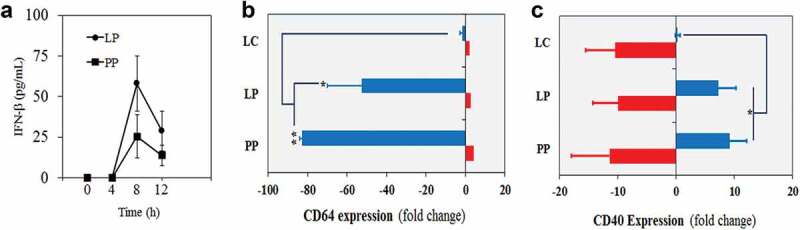
*Lactobacillus plantarum* (LP) and *Pediococcus pentosaceus* (PP) induce responses associated with IFN-I production in monocytes and macrophages from PBMCs. (a) Production of IFN-β in supernatants obtained from primary macrophages after 0, 4, 8 and 12 h of being exposed to LP and PP. The INF-β levels were calculated using the Human IFN-β Quantikine ELISA Kit. Data are representative of three healthy donors and are mean with SD from two biological replicates. (b-c) Expression of (b) CD64 and (c) CD40 in monocytes (blue) and neutrophils (red) exposed to LP and PP as well as *L. casei* (LC) as a control (no IFN-I inducer). The CD expression is presented as a percentage of fold change (increase or decrease) over a non-stimulated condition using unchallenged monocytes and neutrophils. Data are mean with SD from two healthy donors and the comparative analysis was carried out with two-way ANOVA and Tukey multiple comparison (*p < .05; **p < .01).

Next we sought confirmation of LAB-induced IFN-I activation in human primary cells. We first employed an IFN-β ELISA to detect and quantify the presence of this cytokine in the supernatants of human monocyte-derived macrophages collected from PBMC and exposed to *L. plantarum* and *P. pentosaceus* for 2 h. We observed a peak of IFN-β production 8 h post-challenge that decreased at 12 h (Figure 3(a)). We then used flow cytometry to monitor the expression of the IFN-I-related markers CD64 and CD40^35-37^ in monocytes collected from PBMC from two healthy donors upon exposure to LAB. IFN-I expression has been shown to down-regulate CD64^37^ and upregulate CD40 in the presence of LAB.^36^ In agreement with this, we observed that only monocytes exposed to IFN-I-producing *L. plantarum* and *P. pentosaceus* showed a very significant reduction in CD64 expression (Figure 3(b)) and an increase in CD40 expression (Figure 3(c)). No significant changes were observed in the presence of *L. casei*, an isolate that showed a potent NF-κB response but poor IFN-I activation in our initial LAB screening. These findings therefore confirmed that LAB can trigger IFN-I responses in human primary immune cells.

### IFN-I-inducing LAB interact strongly with human monocytes and macrophages

**Figure 4.**
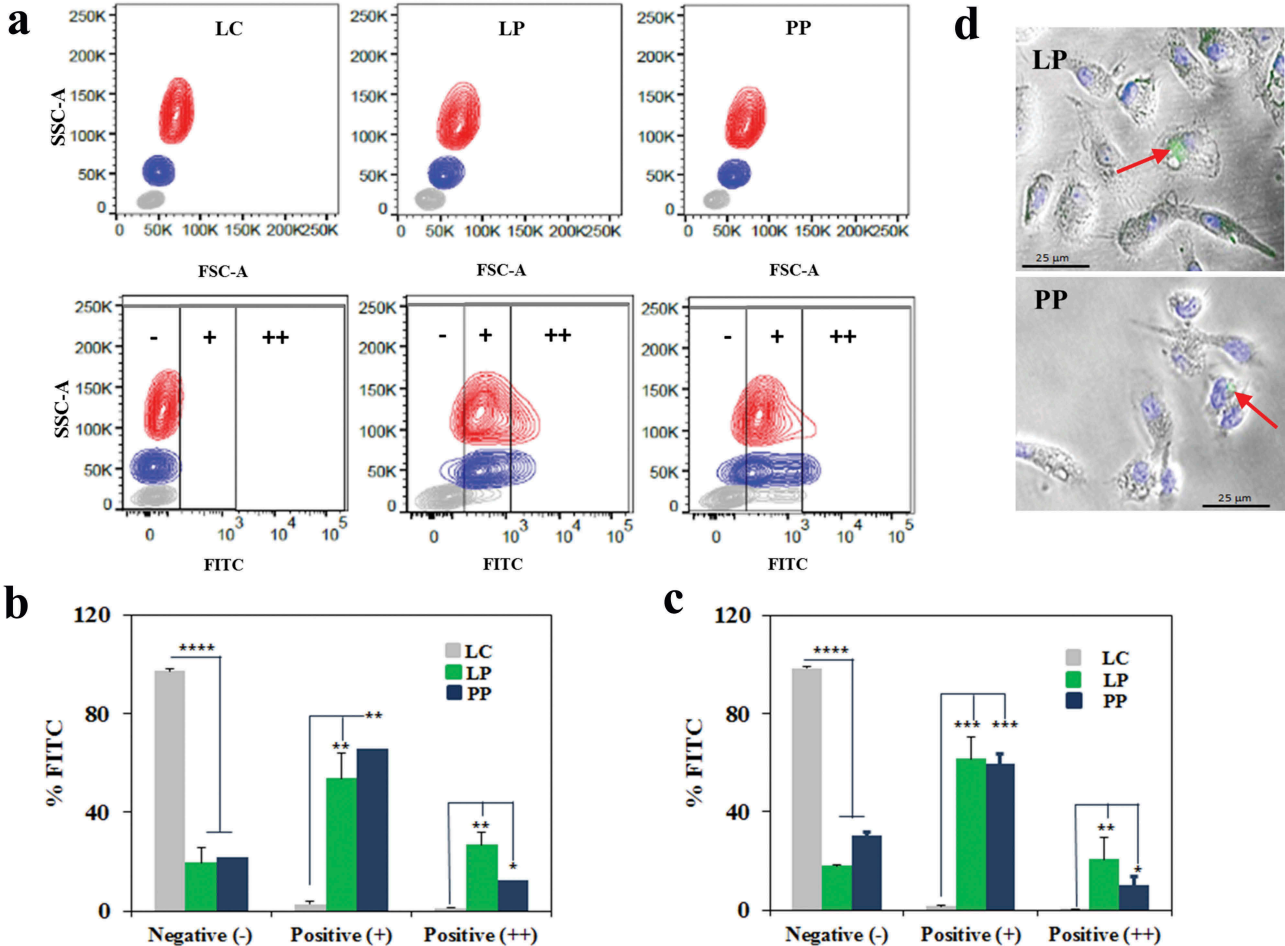
Human phagocytes from PBMCs interact with *Lactobacillus plantarum* (LP) and *Pediococcus pentosaceus* (PP). (a) Interaction of LP and PP as well as *L. casei* (LC) as a control with monocytes and neutrophils from PBMCs of two healthy donors. Viable bacterial cells were labeled with FITC and incubated in whole human blood for 1 h at a ratio of 1:25. Blood cell populations were distinguished based on side scatter area (SSC-A) versus forward scatter area (FSC-A), and separated into lymphocytes (gray), monocytes (blue) and neutrophils (red). Interaction with phagocytes was then observed in the FITC channel, where the intensity was divided into three subpopulations based on the positivity: no interaction (-), binding and/or uptake (+), and strong binding and/or uptake (++). (b-c) Percentage of (b) monocytes and (c) neutrophils in each of the three FITC subpopulations [no interaction (-), binding and/or uptake (+), and strong binding and/or uptake (++)] after exposure to LC (gray), LP (green) and PP (blue). The comparative analysis was carried out with two-way ANOVA and Tukey multiple comparisons (*p < .05; **p < .01; ***p < .005; ****p < .001). (d) Confocal microscopy images showing the detection of LP and PP at the vicinity and/or inside monocyte-derived macrophages (indicated with arrows). FITC and DAPI were used to label bacteria and the macrophage nucleus, respectively.

**Figure 5.**
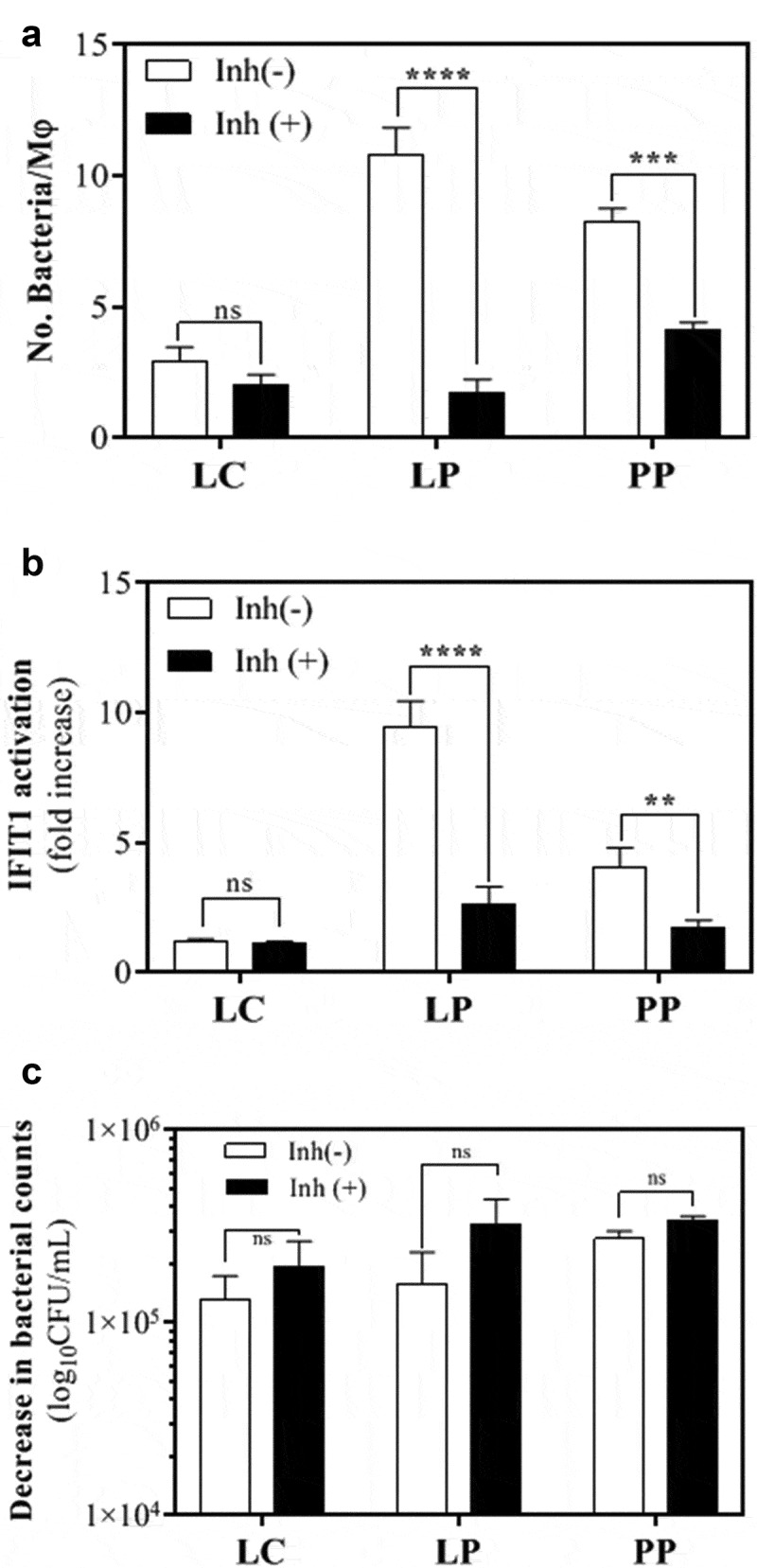
Human THP-1 macrophages interact with *Lactobacillus plantarum* (LP) and *Pediococcus pentosaceus* (PP). (a) Number of *L. casei* (LC), LP or PP cells that were internalized and/or attached to THP1 macrophages after a 2 h RPMI incubation in the absence (white) or presence (black) of the phagocytosis inhibitor cytochalasin D. The bacterial intake and/or attachment was calculated as the difference between the number of bacteria found in the supernatant of THP-1 cells at 0 and 2 h post-LAB challenge, and presented as number of bacteria per macrophage (Mφ). Comparative analysis was carried out using the Student *t*-test (ns, no significant; ***p < .005; ****p < .001). (b) IFIT1 activation in PMA-differentiated IFIT1-GLuc THP-1 macrophages exposed to LC, LP or PP in the absence (white) or presence (black) of cytochalasin D. The activation of IFIT1 is presented as a fold increase over a non-stimulated condition. The bacterial challenge was carried out at a ratio of 1 macrophage per 25 bacteria (**p < .01; ****p < .001; Student’s *t*-test). (c) Decrease in bacterial counts for LC, LP and PP after a 2 h RPMI incubation in the absence (white) or presence (black) of cytochalasin D. The bacterial decrease was calculated as the difference between the number of viable bacteria after 2 h of incubation and expressed as log_10_CFU/mL (ns, no significant; ***p < .005, ****p < .001; Student’s *t*-test).

We next determined the mechanism by which some LAB induce IFN-I production. First, we examined whether *L. plantarum* and *P. pentosaceus* interact with and/or are phagocytosed by human phagocytes (monocytes and macrophages). We carried out three independent assays using PBMCs, macrophages differentiated from PBMCs and THP1 macrophages, at a ratio of 1 phagocyte per 25 bacteria. This ratio ensures a maximum activation of IFIT1 in phagocytes exposed to either *L. plantarum* or *P. pentosaceus* (Figure 1(c)). Firstly, we incubated FITC-labeled LAB with human PBMC from healthy donors for 2 h and then processed the samples by flow cytometry. We identified three populations consisting of leukocytes, neutrophils and monocytes (Figure 4(a), top panels) and then assessed which of these had become positive for FITC (bottom panels). This assay showed that monocytes and neutrophils (blue and red populations) shifted significantly in the presence of *L. plantarum* and *P. pentosaceus*, but not with the NF-κB inducer *L. casei*. We quantitated these data and observed that contrary to *L. casei*, both *L. plantarum* and *P. pentosaceus* interacted significantly with monocytes (Figure 4(b)) and neutrophils (Figure 4(c)). Secondly, we differentiated PBMC monocytes into macrophages and observed them by confocal microscopy upon incubation with FITC-labeled *L. plantarum* and *P. pentosaceus*. We were able to visualize FITC^+^ cells (Figure 4(d)), indicating that a strong interaction occurred between LAB and primary human macrophages. Finally, we studied the effect of pharmacological inhibition of phagocytosis on the internalization of *L. plantarum* and *P. pentosaceus* in human THP-1 macrophages (Figure 5). We observed that the number of *L. plantarum* and *P. pentosaceus* that remain inside (and/or attached to) THP1 macrophages was significantly lower in the presence of the phagocytosis inhibitor cytochalasin D following a 2 h incubation in RPMI (Figure 5(a)). Concomitant with this decrease, the activation of the IFIT1-GLuc reporter upon exposure to *L. plantarum* and *P. pentosaceus* was significantly reduced in the presence of cytochalasin D (Figure 5(b)). Cytochalasin D did not affect bacterial viability (Figure 5(c)) nor affected reporter activation by unrelated agonists such as LPS (not shown). Taken together, these results strongly suggest that *L. plantarum* and *P. pentosaceus* interact and/or are internalized by human macrophages.

### STING and MAVS sense L. plantarum and P. pentosaceus to activate the IFN-I associated kinase TBK1

**Figure 6.**
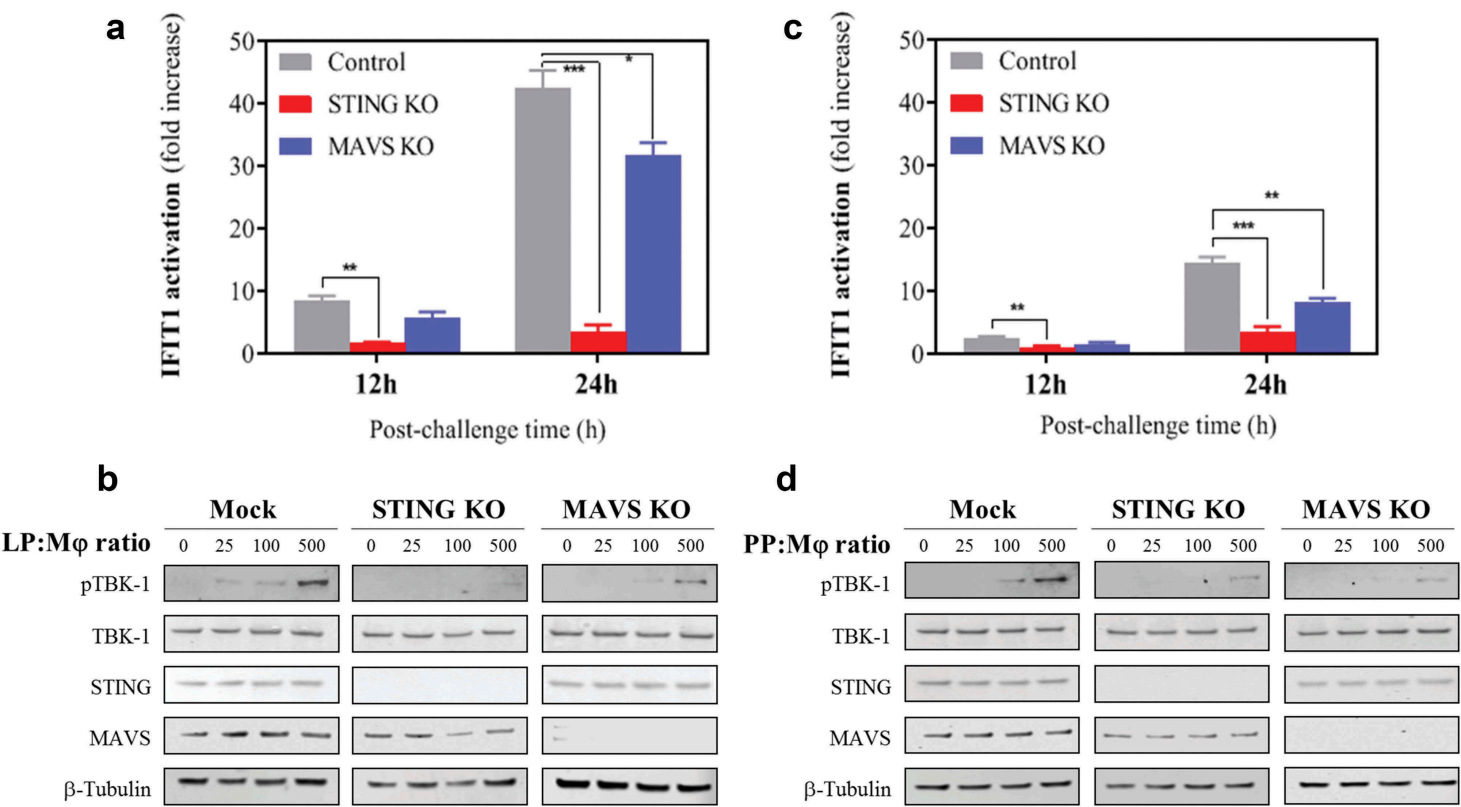
STING and MAVS sense *Lactobacillus plantarum* (LP) and *Pediococcus pentosaceus* (PP), resulting in the activation of the IFN-I associated kinase TBK-1. (a, c) IFIT1-GLuc activation in PMA-differentiated control (gray), STING knock-out (blue) and MAVS knock-out (red) THP-1 cells in response to (a) LP or (c) PP. IFIT1 activation is presented as a fold increase over a non-stimulated condition after 12 and 24 h of incubation using a ratio of 1 macrophage per 25 viable bacteria. For each time point, a comparative statistical analysis was carried out between the control and the knock-out cells using the Student’s *t*-test (*p < .05; **p < .01; ***p < .005). (b-d) Immunoblotting against the indicated proteins in whole-cell lysates from PMA-differentiated control, STING and MAVS knock-out THP-1 cells exposed to (b) LP or (d) PP at the indicated doses.

STING and MAVS are potent IFN-I inducers that respond to cytosolic MAMPs. Having observed that IFN-I-inducing LAB were internalized by macrophages, we explored whether these cytosolic sensors could account for the observed immune responses. We then used THP-1-IFIT1-GLuc cells deficient for STING (STING KO) or MAVS (MAVS KO).^38^ We differentiated these cells into macrophages and challenged them with viable cells of *L. plantarum* and *P. pentosaceus* before measuring GLuc activity. As expected, control cells responded strongly to *L. plantarum* and IFIT1 activation reached 10 and 40 fold increase at 12 and 24 h after challenge. However, this response was completely abrogated in STING KO cells (Figure 6(a)). The response in MAVS KO cells was also reduced, but this was less evident, being only statistically significant after 24 h of bacterial challenge. To verify the role of STING in *L. plantarum*-induced IFIT-1 activation, we examined the levels of phosphorylated TBK-1 in the lysates of cells exposed to different *L. plantarum* doses. Phosphorylated TBK-1 was absent in mock-challenged cells, but was readily detected 2 h after bacterial challenge in a dose-dependent manner (Figure 6(b)). In the absence of STING, we barely detected any phosphorylated TBK-1, whilst in the absence of MAVS, a reduction was appreciated in agreement with the reporter data. We then assessed the response to *P. pentosaceus*. As shown previously, exposure to *P. pentosaceus* triggered a lower response than that to *L. plantarum* and control cells showed elevated luciferase activity at 24 h post-challenge (Figure 6(c)). However, this was also drastically reduced in STING KO cells and partially reduced in the absence of MAVS (Figure 6(c)). Accordingly, levels of phosphorylated TBK1 were also diminished in the absence of STING or MAVS (Figure 6(d)). Collectively, our results demonstrate that *L. plantarum* and *P. pentosaceus* are sensed by mechanisms that involve the intracellular sensors STING and, to a lesser extent, MAVS.

### STING and MAVS are required to induce IFN-I in response to LAB

**Figure 7.**
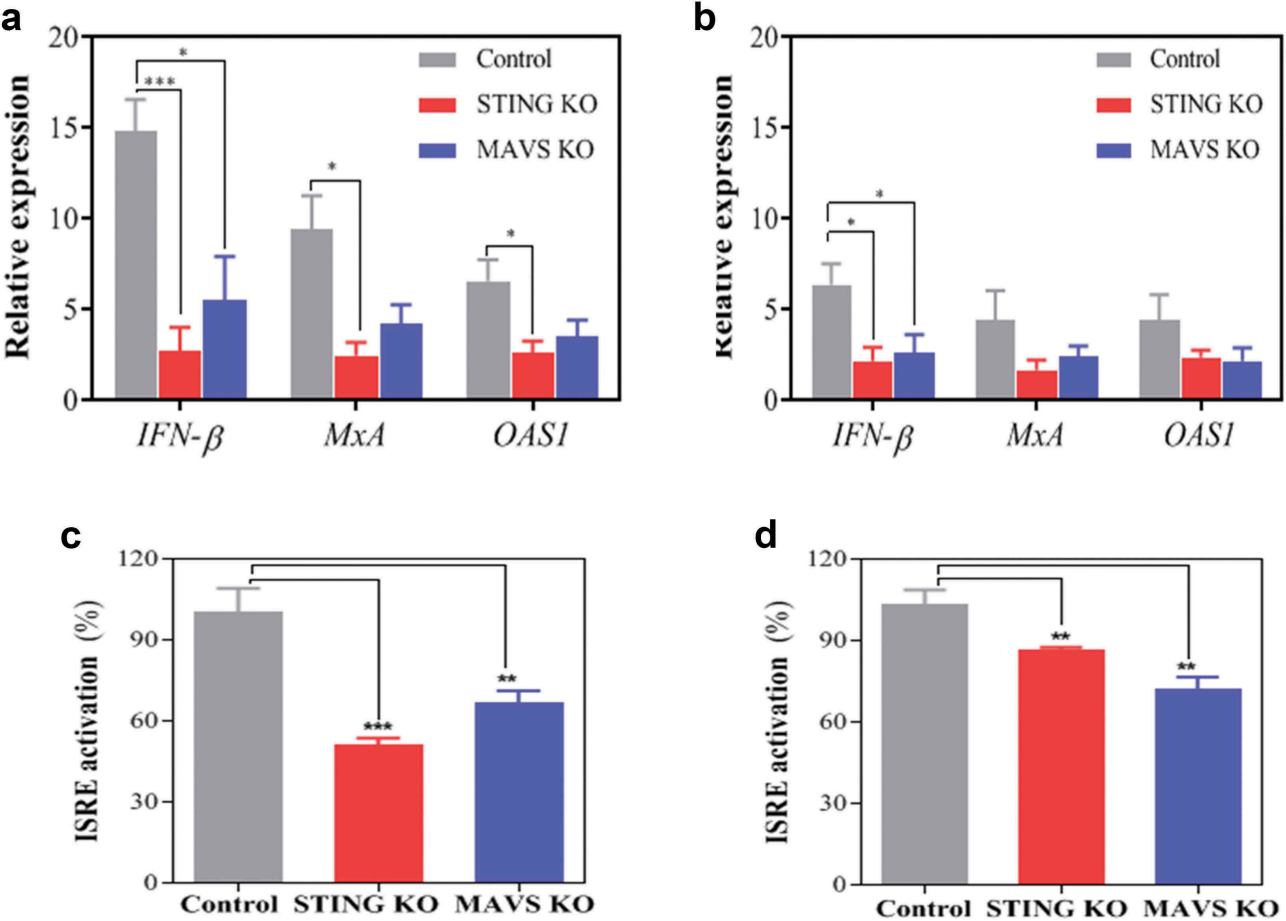
The IFN-I response of macrophages to *Lactobacillus plantarum* (LP) and *Pediococcus pentosaceus* (PP) is dependent on STING and MAVS. (a, b) Level of relative mRNA expression of *IFN-β, MxA* and *OAS1* in IFIT1-GLuc THP-1 macrophages (control, gray) and their corresponding knockouts (KO) for STING (red) and MAVS (blue) after exposure to (a) LP or (b) PP for 8 h at a ratio of 1 macrophage per 25 viable bacteria. The relative expression of each gene is presented as a fold induction following normalization with the internal control gene TBP and nonstimulated THP-1 macrophages. A comparative statistical analysis was carried out between the control and the knockout cells using the Student’s *t*-test (*p < .05, ***p < .005). (C, D) ISRE activation from supernatants obtained from IFIT1-GLuc THP-1 macrophages (control, gray) and their corresponding knockouts (KO) for STING (red) and MAVS (blue) after exposure to (c) LP or (PP) for 24 h at a ratio of 1 macrophage per 25 viable bacteria. The ISRE activation for each cell line was calculated as a fold increase using a pISRE-FLuc/RLuc reporter cell line after 10 h of supernatant exposure and then indicated as a percentage relative to control. A comparative statistical analysis was carried out between the control and the knockout cells using the Student’s *t*-test (**p < .01; ***p < .005).

To evaluate the influence of STING and MAVS on the ability of *L. plantarum* and *P. pentosaceus* to activate IFN-I responses, we determined the induction of IFN-I and IFN-I-associated genes from STING and MAVS KO macrophages exposed to *L. plantarum* and *P. pentosaceus* at a ratio of 25 bacteria per macrophage (Figure 7). We first measured the relative expression of *IFN-β, MxA* and *OAS1* in the challenged KO macrophages (Figure 7(a,b)). After 8 h of bacterial challenge with *L. plantarum* we detected a significant increase in the mRNA expression of *IFN-β* in the control cells (Figure 7(a)). In both STING and MAVS KO cells, this increase was significantly lower, in particular in the STING KO cells. Similarly, the absence of STING and MAVS in cells previously exposed to *L. plantarum* resulted in a significant decrease in the expression levels of *MxA* and *OAS1*, two well-known ISGs. The mRNA expression of these two ISGs, as well as IFN-β, also decreased in STING KO and MAVS KO cells challenged with *P. pentosaceus* as compared with the control cells (Figure 7(b)). However, only the reduction in IFN-β expression was found to be statistically significant. Finally, media from cells exposed to *L. plantarum* (the most potent inducer of IFN-I) were subjected to ISRE bioassay. In agreement with gene expression data, absence of STING and, to a lesser extent MAVS, significantly reduced the amount of biologically active IFN-I (Figure 7(c)). We observed similar results with KO cells exposed to *P. pentosaceus* but the reduction in functional IFN-I was less clear and equally significant from both KO cells (Figure 7(d)).

## Discussion

Human cells express multiple PRRs aimed at detecting the presence of foreign molecules including those from bacteria and viruses. Such recognition activates signaling mechanisms that converge in the production of cytokines and chemokines. Currently, the central dogma is that commensal species are sensed by TLRs and NLRs and trigger activation of NF-κB. Certainly, LAB, a major commensal group, have been shown to activate NF-κB via TLR2,^39^ TLR9^40^ and NLRs.^41^ A few studies have also shown that LAB are capable of inducing the production of IFN-I in innate immune cells,^23,24,42,43^ and when the mechanism has been reported, this has been linked with TLR2/3 recognition via endosomes.^23,24^ In this study, we report the production of IFN-I by LAB in human macrophages and demonstrate that such production is mediated by intracellular sensing driven by the nucleic acid sensors STING and MAVS. Our findings were generated from an initial-unbiased screen using different representative LAB species. This screen showed that macrophage challenge with LAB species such as *E. faecalis, L. casei, L. sakei* and *P. acidilactici* resulted in very high NF-κB activation, as widely reported in previous publications.^44–47^ However, unlike most of the selected LAB, *L. plantarum* and *P. pentosaceus* failed to significantly activate the NF-κB pathway, but were instead able to induce production of IFN-I in human macrophage-like cells and human primary phagocytes isolated from PBMCs. LAB induction of IFN-I production was species-specific since it was not observed for other lactobacilli and pediococci tested in our screen. This bacterial specificity has been confirmed with other strains of *L. plantarum* and *P. pediococcus* that we have recently isolated from animals.^47,48^ The level of IFN-I produced by these bacteria in macrophages was detectable by ELISA, but more importantly, it induced upregulation of CD40 and downregulation of CD64, both of which are well-known IFN-I-responsive markers.^35–37^ In this respect, Weiss *et al*. found that LAB that induce IFN-β activation are also able to stimulate CD40 in murine bone marrow derived dendritic cells,^36^ a positive convergence that we have confirmed here using human primary monocyte-derived macrophages. Furthermore, IFN-I triggered the production of ISGs in the form of *MxA* and *OAS1*, effectively activating an antiviral response. Our work therefore may provide explanations for how the composition of microbial communities and specific bacterial groups modulate innate immune responses during virus infection,^49^ an emerging field with impact on gastrointestinal and non-gastrointestinal infection as well as vaccines.

The IFN-I activation that we have recorded here required live bacteria and their interaction with paghocytes, as previously reported with dentritic cells stimulated with other LAB species.^23^ Alive cells of *L. plantarum* and *P. penstosaceus* triggered an endogenous IFN-I production that was significantly higher than that observed with inactivated cells. In general, macrophages exposed to heat-killed (or inactivated) bacterial cells activate TRL/NOD-like receptors-dependent pathways, whereas live bacteria activate other pathways that require phagocytosis and bacterial degradation in the phagolysosome, which may lead to the release of bacterial intracellular components into the cytosol.^50^ Recently, it has been shown that STING is essential to activate an integrated stress response to live Gram-positive bacteria particularly *Listeria*, and that part of this response involves the late production of IFN-I.^18^ STING activation occurred upon engagement with the CDN c-di-AMP secreted by these bacteria and therefore it bypassed cGAS.^18,51,52^ Whilst many clinically relevant Gram-positive bacteria including *L. monocytogenes* or *Mycobacterium tuberculosis* produce c-di-AMP and are hence susceptible to STING responses, it is unclear whether this is also the case for LAB. The synthesis of CDNs has been described in LAB, but very little is known about their role in bacterial physiology and host innate immune responses.^53^ More recent work has identified the oxidoreductase RECON as a high-affinity cytosolic sensor of CDNs including c-di-AMP,^54^ and it has been proposed that bacteria such as *L. monocytogenes* secrete c-di-AMP to inhibit RECON and relieve RECON-mediated NF-κB inhibition to promote bacterial spread.^55^ Therefore, if LAB were inducing IFN-I via c-di-AMP secretion, a concomitant increase in NF-κB response would have been expected. Our findings indicating that IFN-I-inducing LAB hardly activated NF-κB suggest that other PAMPs and mechanisms may be responsible for STING sensing. A possible explanation to account for the observed STING-dependent responses would be the release of bacterial DNA into the cytosolic millieu acting as an intracellular PAMP. We believe that the requirement for live LAB cells could be associated to the process of internalization and subsequent DNA recognition by professional phagocytes.

Recent studies have reported that the cytosolic sensors STING and MAVS are important to maintain gut homeostasis and prevent autoimmune conditions such as inflammatory bowel disease (IBD).^56,57^ Our results are in line with these latest discoveries. Although multiple commensals might be involved in beneficial nucleic acid responses in the gut, our study has identified *L. plantarum* as a potential key player. We have proved that *L. plantarum* interacts intimately with human phagocytes and that this close interaction is essential to activate IFN-I responses via STING and MAVS. We have also observed that *L. plantarum* failed to activate NF-κB, a transcription factor that drives pathology in many inflammatory disorders. In these, microbial dysbiosis and the continuous production of potent NF-κB-mediated inflammatory cytokines such as TNF-α drive perpetual inflammation. IFN-I is emerging as an important cytoprotective signature in mucosal tissues including the gut.^58,59^ Therefore, *L. plantarum* and their mechanisms could assist in targeting the underlying microbe-immune cell interactions and inducing beneficial anti-inflammatory cytokines. *L. plantarum* is a frequent inhabitant of mucosal tissues where a variety of immune cells including macrophages and dendritic cells lie and it is widely recognized as a potential immunomodulator. At present, it is unclear how *L. plantarum* is recognized by macrophage receptors and what properties this bacterium has to account for its ability to activate STING and MAVS. Our results comparing phagocytic uptake of *L. plantarum* with other LAB such as *L. casei* (Figure 4) indicates that the level of interaction with human phagocytes might be a prominent differential feature, which eventually leads to phagocytic uptake and intracellular processing of the bacterial cells. Although multiple factors may exist, one possibility explaining this enhanced interacting capacity is the ability of *L. plantarum* to express adhesins that facilitate interactions with other cells.^60^ The oral administration of *L. plantarum* as a probiotic is common and remarkably it has resulted in a significant reduction in respiratory tract infections in newborns.^61^ These probiotic features and the mechanisms underlying the IFN-I activation that we report in this work indicate that *L. plantarum* might be an excellent candidate to mitigate inflammation as well as enhancing protective antiviral responses.

In summary, our findings highlight the value of LAB and their molecules as potential mediators of host protective IFN-I responses via the cytosolic sensors STING and MAVS. A better understanding of the STING-mediated IFN-I regulation by *L. plantarum* and other LAB that have a very low impact on NF-κB-mediated pro-inflammatory cytokines could be helpful in the design of future probiotic therapies addressing human gut disorders.

## Materials and methods

### Ethics statement

All procedures with human blood samples have been approved by the Surrey University Ethics Committee in accordance with the Institutional Policy on the Donation and Use of Human Specimens in Teaching and Research and the national guidelines under which the institution operates. The blood protocol approval was under IRAS number 236477 and the sample storage was carried out under HTA license 12365.

### Human peripheral blood mononuclear cells (PBMC) and macrophages

Whole fresh blood was collected in heparinized tubes from healthy individuals to carry out the phagocytosis assay. To generate macrophages, monocytes were first isolated from buffy coats of the healthy blood donors by Lymphoprep (Axis-Shield) density gradient centrifugation followed by plastic adherence in Roswell Park Memorial Institute (RPMI) 1640 (Life Technologies) supplemented with 15% fetal bovine serum (FCS, Seralab) and 1% Penicillin/Streptomycin (Pen/Strep, Life Technologies) at 37°C in an atmosphere of 5% CO2. The collected peripheral blood mononuclear cells (PBMCs) were incubated for 2 h to remove nonadherent cells by washing with PBS. The adherent monocytes were then detached using 10 mM EDTA in PBS at room temperature and plated for macrophage maturation in RPMI with FCS and Pen/Strep for 7 days.

### THP-1 cell lines and macrophage differentiation

THP-1 cells were propagated in RPMI supplemented with 15% FCS and 1% Pen/Strep. THP-1-κB-GLuc (Lucia™ NF-κB) express and secrete Gaussia luciferase (GLuc) under the control of a synthetic 5x-κB promoter (Invivogen). THP-1-IFIT1-GLuc express and secrete Gluc under the control of the promoter of the IRF-3-dependent gene IFIT1 and were a gift from Veit Hornung.^62^ THP-1-IFIT1 deficient for STING or MAVS, and its corresponding control, were kindly provided by Greg Towers and have been previously described.^38^ THP-1 monocytes were differentiated into macrophages in RPMI supplemented with 20 ng/mL of phorbol 12-myristate 13-acetate (PMA) for 48 h as previously described.^63^ After the 48 h incubation medium was replaced for RPMI containing 2% FCS and 1% Pen/Strep and macrophages exposed to LAB. PMA was from Santa Cruz Biotechnology and kept in DMSO at 10 mg/mL.

### Growth and maintenance of LAB

The LAB selected for this study is shown in Table 1 and are reference strains with accessible peer-reviewed publications and/or genome sequences with the exception of *Pedioccocus acidilactici* (isolate E1) and *Lactobacillus kunkeei* (isolate O29). The genomes of the newly-characterized isolates E1 and O29 and their corresponding sequencing reads, assemblies and metadata have been uploaded onto Genbank in BioProject PRJNA544274. All the isolates were grown in MRS broth (Oxoid) at 37ºC without any aeration for 24 h, and maintained as −80°C frozen stocks in their appropriate media with the addition of 15% glycerol.

### Bacterial challenge

PMA-differentiated THP-1-NF-κB-GLuc and THP-1-IFIT1-GLuc macrophages were exposed to inactivated and viable LAB cells. The inactivated cells were used as heat-treated (70°C, 2 h) LAB pellets at a ratio of 100 cells per 1 macrophage and incubated for 24 h, while viable cells were incubated simultaneously with the macrophages for 2 h in RPMI without Pen/Strep at different doses as indicated in the figure legends before being replaced for fresh RPMI with antibiotics to incubate for a further 22 h. To measure Gluc activity, media were collected from the macrophage cultures and transferred to white-bottom 96-well plates, which were read in a Clariostar plate reader (BMG Biotech) in the presence of 2 μg/mL of coelenterazine (NanoLight Technology). LPS (Sigma Aldrich) was used at 0.2 mg/ml as a control for the activation of NF-κB and IFIT1. Activation of NF-κB or IFIT1 was calculated as a fold increase ± SD over the measurements recorded for unchallenged macrophages.

### Bacterial interaction with THP-1 macrophages

In order to estimate the number of LAB that interact with and/or are internalized by THP-1 macrophages, we carried out an extracellular survival assay as previously described^64^ with minor modifications. Macrophages were incubated with viable LAB at a ratio of 25 bacteria per phagocyte in RPMI containing 2% FCS for 2 h, at 37°C and 5% CO_2_. Macrophages were treated with the phagocytosis inhibitor cytochalasin D at a final concentration of 10 μM (Sigma-Aldrich) and subsequently challenged with LAB. LAB were also incubated without macrophages under the same experimental conditions (with or without cytochalasin D). Supernatants were then collected at time points 0 and 2 h to calculate and compare the number of bacteria that remained viable and detached from THP-1 macrophages. Bacterial enumeration was performed by quantitative plating of serial dilutions on MRS agar plates.

### Phagocyte assay with human peripheral blood mononuclear cells (PBMC)

Approximately 10^6^ leukocytes were mixed 1:1 with 10 mM EDTA in PBS and challenged with LAB that were previously labeled with the FITC dye (Sigma), at a ratio of 25 bacteria per cell. The phagocytosis incubation was then carried out at 37°C in an orbital shaker for 1 h and subsequently treated with 1x RBC lysis solution (Biolegend) following incubation at room temperature for 15 min. The cells were washed twice with 10nM EDTA in PBS, resuspended in PBS and analyzed on FACS Celesta instrument (BD Biosciences). Flow cytometry analysis based on forward (FSC) and side (SSC) scatter was used to distinguish the main blood cell populations based on their size and granularity (lymphocytes vs. phagocytes), while the FITC channel was used to measure bacterial interaction with blood cells. The resulting SCC/FITC plots were then used to quantify the LAB intake by phagocytes.

### CD expression in phagocytes from human PBMCs

In order to monitor the expression of CD40 and CD64 in monocytes and neutrophils exposed to LAB we carried out the phagocytosis assay as described above but with some modifications. Leukocytes and bacteria were mixed at 1:100 ratios and incubated for 1 h. After phagocytosis incubation antibodies against human CD40-BV510 and CD64-PE/Dazzle (Biolegend), or the corresponding isotype controls, were added to the samples and incubated for 20 min at room temperature. The fixable viability dye Zombie NIR (Biolegend) was also used for a live/dead stain as per manufacturer’s instructions. Cells were then fixed with RBC lysis buffer and the CD expression compared to the matching isotype control and no-bacteria controls to calculate expression levels in each of the blood cells populations identified by FACS.

### Microscopy studies on monocyte-derived macrophages from human PBMCs

Monocyte-derived macrophages were challenged for 2 h with FITC-labeled LAB as described above for THP-1 cells. After phagocytosis macrophages were fixed for 20 min with 1% paraformaldehyde (PFA), washed with PBS without Calcium or Magnesium and stained with DAPI for imaging on an EVOS fluorescent microscopy.

### SDS-PAGE and immunoblotting

LAB-challenged THP-1 cells were lysed in radio-immuno-precipitation assay (RIPA) buffer supplemented with protease and phosphatase inhibitors (Roche) and benzonase at 250 U/ml (Sigma). Lysates were rotated for 30 min at 4°C and subsequently denatured for 5 min at 95°C in the presence of loading buffer and resolved by SDS-PAGE. The samples were then transferred to nitrocellulose membranes (GE Healthcare) using a Trans-Blot semidry transfer unit (Bio-Rad). Membranes were blocked in 0.1% Tween/phosphate-buffered saline supplemented with 5%-skimmed milk (Sigma) and subjected to immunoblotting with the following primary antibodies at the indicated dilutions: phosphorylated TBK-1 Ser^17^ (Abcam; 1:5,000), TBK−1 (Abcam; 1:5,000) and α-tubulin (Upstate Biotech; 1:10,000). Primary antibodies were detected using IRDye-conjugated secondary antibodies in an Odyssey infrared imager (LI-COR Biosciences). Images were analyzed using Odyssey software.

### Quantitative RT-PCR

PMA-differentiated THP−1 cells were exposed to LAB for 8 h and cells were collected for RNA extraction using the High Pure RNA Isolation Kit (Roche Diagnostics Limited) following manufacturer’s instructions. RNA was reverse transcribed using the SuperScript ® III First-Strand Synthesis System from Invitrogen and the resulting cDNA diluted 1:5 in water. cDNA was then used as a template for real-time amplification on a QuantStudio 7 Flex Real-Time PCR system (Applied Biosystems) using SYBR Green Master Mix (Applied Biosystems) and specific primers for: TATA box Binding Protein (TBP) (forward, 5ʹTGCACAGGAGCCAAGAGTGAA; reverse, 5ʹ CACATCACAGCTCCCCACCA), myxovirus resistance gene A (MxA) (forward, 5ʹ CCCCAGTAATGTGGACATCG; reverse, 5ʹ ACCTTGTCTTCAGTTCCTTTGT), human 2ʹ5’-oligoadenylate synthetase 1 (OAS1) (forward, 5ʹ TGTGTGTGTCCAAGGTGGTA; reverse, 5ʹ TGATCCTGAAAAGTGGTGAGAG), and human IFNβ (described previously^65^) using three technical replicates of samples obtained from three biological replicates. Expression of each gene was normalized to the house-keeping gene TBP, and these values were then normalized to the non-stimulated control cells to yield a fold induction.

### ISRE-based IFN-I bioassay

Cell culture supernatants from LAB–challenged, PMA-differentiated THP-1 macrophages were collected after 24 h of incubation and transferred to 96-well plates containing HEK293T cells previously transfected for 24 h with 70 ng/well of a reporter plasmid expressing firefly luciferase (FLuc) under the control of ISRE and 10 ng/well of a control plasmid expressing *Renilla* luciferase (RLuc) (Promega) using polyethylenimine (PEI; Sigma) at a ratio of 1:2 (μg of DNA:μl of PEI). After 10 h the cells were lysed in passive lysis buffer (Promega) and the FLuc and RLuc activities measured in the Clariostar plate reader (BMG Biotech). The FLuc/RLuc ratios were then calculated for each well and normalized to mock-infected THP-1 samples to be presented as a fold increase of ISRE activation.

### ELISA cytokine detection

Human TNF-α production was detected and quantified in the supernatants of LAB-challenged PMA-differentiated THP-1 cells using the eBioscience Human TNF-α ELISA Ready-SET-Go kit as indicated by the manufacturer’s instructions. The Human IFN-β Quantikine ELISA Kit (R&D systems) was used to detect and quantify IFN-β in supernatants from primary macrophages exposed to LAB for 2 h. Supernatants were collected every 4 h for 12 h after the exposure to LAB.

### Statistical analysis

Statistical analysis was performed using GraphPad Prism. Data are presented as means ± standard deviation (SD) and are representative of one experiment of at least three independent experiments. Data from experiments with human peripheral blood mononuclear cells are representative of two or three healthy donors and are mean with SD from two biological replicates. Statistical significance between one sample and its corresponding control was determined using the Student’s *t*-test and within a group of samples using one way ANOVA followed by Fisher’s Least Significant Difference (LSD) Test.
